# The Invasion and Metastasis Promotion Role of CD97 Small Isoform in Gastric Carcinoma

**DOI:** 10.1371/journal.pone.0039989

**Published:** 2012-06-29

**Authors:** Daren Liu, Bogusz Trojanowicz, Longyun Ye, Chao Li, Luqing Zhang, Xiaowen Li, Guogang Li, Yixiong Zheng, Li Chen

**Affiliations:** 1 Department of Surgery, Second Affiliated Hospital, College of Medicine, Zhejiang University, Hangzhou, People’s Republic of China; 2 Forschungs-Labor, Klinik für Innere Medizin II, Universitätsklinikum Halle(Saale), Halle (Saale), Germany; Blaise Pascal University, France

## Abstract

CD97 is over-expressed in the majority of gastric adenocarcinomas and is associated with its dedifferentiation and aggressiveness. Our previous results demonstrated that out of three CD97 isoforms tested, only the small one was able to promote increased invasiveness *in vitro*. Based on these data we further aimed to investigate the role of CD97 small isoform in gastric cancer progression *in vivo* by employing the cells with a stable CD97 small isoform knock-down and an orthotopic gastric cancer mouse model. We could demonstrate that the knock down of CD97/EGF1,2,5, led to a significant decrease in the number of cells penetrating the gelatin coated membrane as compared with control cells. In the gastric cancer mouse model, both the hypodermic and the orthotopic yielded tumor masses of the CD97/EGF1,2,5kd group and were significantly smaller than the control. Metastatic tumor cell number in early metastatic regional lymph nodes on post-operative day 42 was distinctly decreased in the CD97/EGF1,2,5kd group as compared with the SGC-NS group, and was accompanied with the downregulation of CD44, VEGFR, CD31 and CD97. We concluded in this study that CD97 small isoform not only supported gastric cancer local growth, but also promoted metastatic spread in orthotopically implanted mouse model suggesting involvement of the CD97 small isoform in the preparation of (pre)metastatic niche.

## Introduction

CD97 is a member of a new subgroup of seven-span transmembrane (TM7) molecules which belong to the secretin receptor superfamily [1]–[3]. CD97 is produced as alternatively spliced forms that contain three (EGF1,2,5), four (EGF1,2,3,5), or five (EGF1-5) repeated EGF-like domains which mediate binding to its cellular ligand, decay accelerating factor (DAF, CD55), a regulatory protein of the complement cascade [4]–[6]. CD97 was originally found to be expressed by hematopoietic cells [7], [8], then abundantly detected in various normal tissues and advanced stages of thyroid, colorectal, gastric, pancreatic, esophageal and oral squamous cell carcinomas [9]–[14]. CD97 protein is over-expressed in majority of gastric adenocarcinomas (60%–88%) and mostly located in the tumor cells at the invasion front, which has higher motility as compared to the cells in the solid formation [15], [16]. Various studies reported that elevated expression of CD97 in gastric cancer is associated with the dedifferentiation and aggressiveness of tumor cells and directly correlates with clinicopathological parameters such as TNM classification [14], [16]. Recently, the interaction between the small isoform of CD97 (CD97/EGF1,2,5) and its ligand CD55 led to increased motility and elevated proteolytic activity of matrix metalloproteinases or chemokine secretion was revealed in colorectal cancer cells [17], [18].

Our previous studies revealed that out of three CD97 isoforms, only the small one was associated with increased invasive behavior of gastric cancer cells in vitro [19]. However, knowledge of the role of CD97 isoforms, especially CD97/EGF1,2,5, in tumor metastasis in vivo is still lacking.

In this study, by employing the cells with stable CD97 small isoform knock-down and orthotopic gastric cancer mouse model, we further investigated the role of CD97 small isoform in gastric cancer progression in vivo, focusing on tumor development and metastatic potential.

## Results

### Generation of Transfectants with Stable Knockdown of CD97 Small Isoform

**Figure 1:**
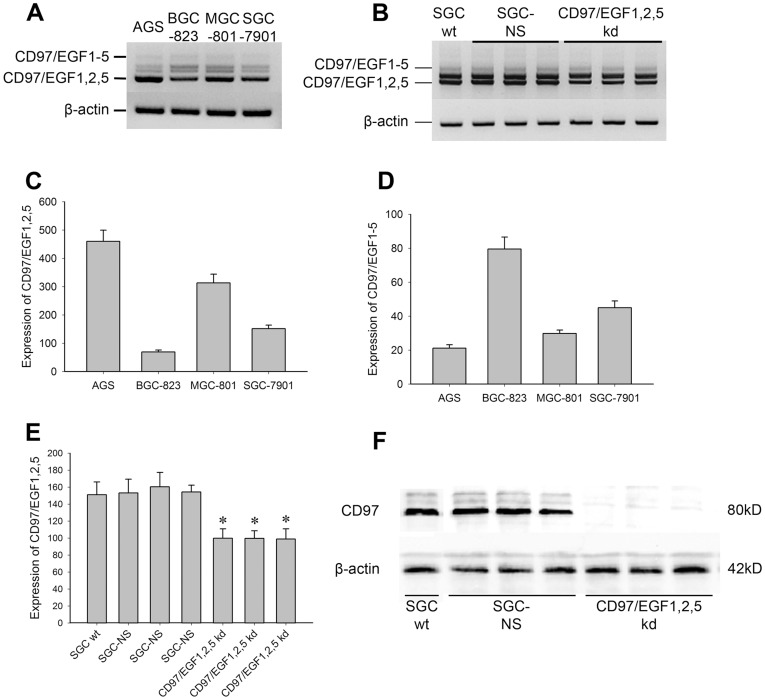
Generation of transfectants with stable knockdown of CD97 small isoform. (A) CD97 expression on gastric carcinoma cell lines SGC-7901, AGS, BGC-823 and MGC-801. (B) Expression of CD97 isoforms in SGCwt, SGC-NS and CD97/EGF1,2,5 knockdown groups. Semi-quantitative RT-PCR analysis, performed with the Bio 1D software, revealed differentially expressed (C) CD97 small and (D) big isoforms of the four cell lines, and significantly decreased levels of (E) CD97 small isoforms in CD97/EGF1,2,5 knockdown group as compared with corresponding controls (*p<0.01). (F) The ∼85 kDa CD97 protein was detected by Western blot analysis in total cellular extracts of SGCwt cells, SGC-NS and CD97/EGF1,2,5 knockdown transfectants.

For this purpose, four human gastric cancer cell lines, SGC-7901, AGS, BGC-823 and MGC-801 were investigated for the CD97 gene expression. RT-PCR revealed that all cell lines investigated expressed CD97, however with different isoform distribution (EGF1,2,5; EGF1,2,3,5 and EGF1-5) (Figure 1A). As demonstrated before [19], BGC-823 cells had the lowest intensity of CD97/EGF1,2,5 but the strongest intensity of CD97/EGF1-5, while AGS showed the strongest expression of CD97 small isoform but the weakest of CD97 big isoform. SGC-7901 which expressed moderate levels of both CD97 small and big isoforms was selected to investigate the specific effects of CD97 small isoform (Figure 1C-D). After we transfected SGCwt cells with shRNAs targeting four different sites of CD97/EGF1,2,5 and selected stable clones by G418, the expression of CD97 small isoform was evaluated by RT-PCR and western blot. As compared with wild-type cells or the clones bearing non-silencing shRNAs, the clones expressing the forth shRNA (CD97iso3-Si4) displayed a 40% loss of CD97/EGF1,2,5 mRNA expression (Figure 1E) and a nearly total loss of CD97 protein (∼80 kDa) (Figure 1F). The silencing of CD97/EGF1,2,5 by employing other sh-RNA site induced similar reduction and effects as compared to CD97iso3-Si4. It is worth to note that due to the knock-down of CD97/EGF1,2,5, the other two isoforms (CD97/EGF1,2,3,5 and CD97/EGF1-5) were not significantly affected.

### Effect of CD97 Small Isoform on Proliferation, Migration and Invasion of Human Gastric Cancer Cells

**Figure 2:**
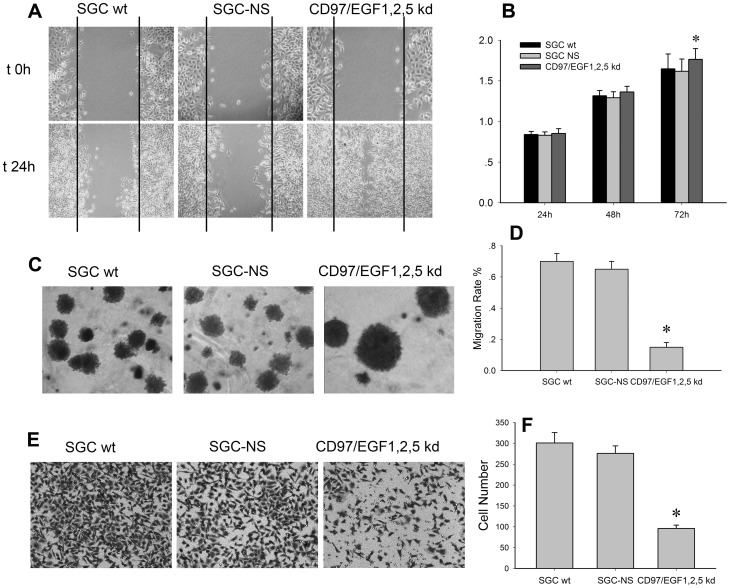
Effect of CD97 small isoform on proliferation, migration and invasion of human gastric cancer cells. The migration ability of SGCwt cells, SGC-NS and CD97/EGF1,2,5 groups were assessed by scratch-wound assay (A) and soft agar (C), which showed bigger size colonies and aggregated type of growth. The migration rate (D) was calculated as the proportion of the mean distance between both borderlines caused by scratching to the distance that remained cell-free after re-growing (*p<0.01). (B) MTT assay revealed the proliferative ability was significantly higher in CD97/EGF1,2,5 kd group as compared to the controls (*p<0.01). (E,F) Invasive assay revealed the number of penetrated CD97 small isoform kd clones was significantly decreased as compared to the control (*p<0.01).

Increased proliferation and migration are both important parameters defining cancer cells and their reductions may serve as potential anti-cancer therapy. In this study, the proliferative ability of CD97/EGF1,2,5 kd clones was significantly higher as compared to the wild-type and control groups, which coincided with our previous research (Figure 2B). Using the scratch-wound assay, a continuous movement was observed in all the three groups, but a more significant movement of the CD97/EGF1,2,5 kd clones migration front was evident at 24 h, in which the cell-free “scratch” region was almost full confluent. Upon comparison with wild-type and empty control groups, the migration of CD97/EGF1,2,5 kd clones was significantly enhanced after 24 h of incubation (P<0.01) (Figure 2A, 2D). These observations revealed that the down regulation of CD97 small isoform did not result in an inhibition of proliferative or migrative ability of gastric cancer cells as previously expected, but caused a statistical significant enhancement of both the proliferative and migrative ability of SGC-7901 cells.

Soft agar colony formation assay revealed the different invasive ability of CD97/EGF1,2,5 kd clones. CD97/EGF1,2,5 kd clones generated 5 times less colonies as compared with wild-type and empty control cells. Furthermore, the colonies formed by CD97/EGF1,2,5 kd clones were bigger in size and showed the preference of aggregated growth rather than the pattern of detachment from the main tumor bulk and disseminated growth (Figure 2C). To confirm whether CD97 small isoform may affect the extracellular matrix by alterations of penetrating ability, transfectants investigated were seeded on filters coated with gelatin. After 24-h incubation in the upper chamber, the cells that had migrated to the other side of the filter were stained and counted. The number of penetrated CD97/EGF1,2,5 kd clones was significantly decreased when compared with empty control, which indicated its partial loss of invasive ability after the knock down of CD97 small isoform (Figure 2E,2F).

### CD97 Small Isoform Supports Gastric Cancer Local Growth

**Figure 3:**
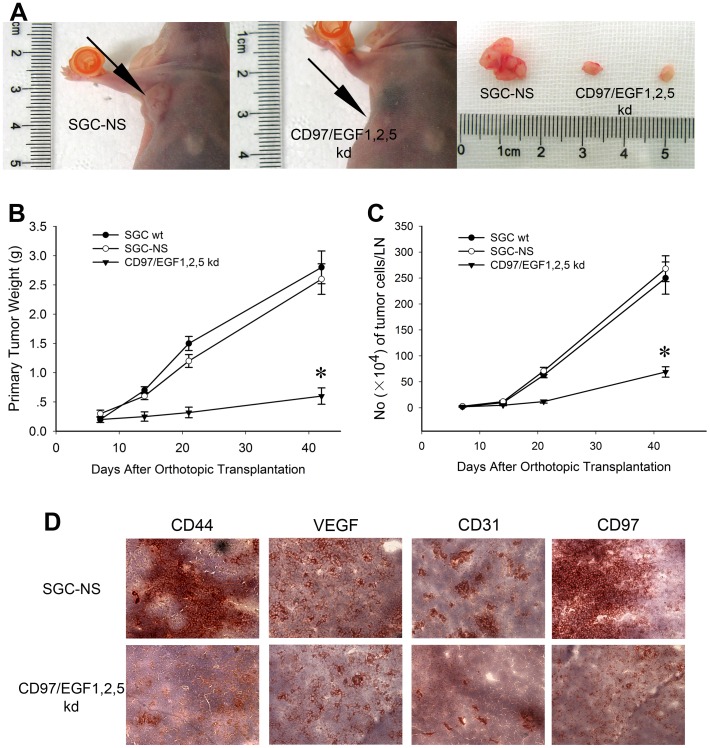
CD97 small isoform supports metastatic spread of orthotopically implanted gastric cancer. (A) The size of subcutaneous formed tumor masses of CD97/EGF1,2,5 kd group was significantly smaller. (B) Primary tumor weight of orthotopically transplanted gastric cancer in CD97/EGF1,2,5 kd group was significantly lighter (*p<0.01). (C) The metastatic tumor cells within regional lymph nodes were counted by C4.4A staining and FACS (*p<0.01). (D) Immunohistochemistry: CD44, VEGFR, CD31 and CD97 expression in early metastatic regional lymph nodes of CD97/EGF1,2,5 kd group were strongly down regulated.

Aiming at verifying the invasion promoting role of CD97 small isoform in vivo, a mouse orthotopic gastric cancer model with a high frequency of lymph node metastasis was employed. Six-eight weeks after the hypodermic inoculation of SGC wt, SGC-NS and CD97/EGF1,2,5 kd clones, 100% of gastric cancer cells (10/10) grew as subcutaneous implants in nude mice. The size of subcutaneous formed tumor masses of the SGC wt (1.05±0.3 cm) and SGC-NS (1.01±0.2 cm) groups were significantly bigger than the CD97/EGF1,2,5 kd group (0.4±0.05 cm) as indicated in Figure 3A and no regional or distant metastasis was observed. Hypodermic tumor masses were harvested and orthotopically transplanted as described. Primary tumor masses and metastases which included local and distant lymph nodes, the liver, the lung and the pancreas were weighted and harvested on post-operative days 7–42 and histologically confirmed. Similar with the hypodermic yielded tumor masses, the weight of primary tumor masses of CD97/EGF1,2,5 kd group (0.6±0.14 g) were significantly lower as compared to SGC wt (2.8±0.26 g) and SGC-NS (2.6±0.28 g) groups on post operative days 42, which again suggested the tumor supporting effect of CD97 small isoform (Figure 3B).

### CD97 Small Isoform Supports Metastatic Spread of Orthotopically Implanted Gastric Cancer

To further evaluate the role of CD97 small isoform in lymph node metastasis, metastatic tumor cells within lymph nodes along the lesser gastric curvature were counted by C4.4A staining and flow cytometry (Figure 3C). Though only small amount of tumor cells were detected on post operative days 7 (2–3±1×10^4^/LN), the frequency of lymph node metastasis was 100% (60/60). The tumor cell number within CD97/EGF1,2,5 kd group (69±10×10^4^/LN) was distinctly decreased on post operative days 42 as compared to SGC wt (250±31×10^4^/LN) and SGC-NS (268±25×10^4^/LN) groups.

Further investigations were performed on EPCAM, VEGFR and CD44, tetraspanins and integrins previously reported to participate in tumor progression by preparation of (pre)metastatic niche. In this study, CD44, VEGFR, CD31 together with CD97 antibodies were employed to examine the metastasis-supporting effect of CD97 small isoform and the alterations of transmembrane receptors protein expression by down regulation of CD97/EGF1,2,5. Strongly down regulated CD44, VEGFR, CD31, as well as CD97 expression in early metastatic regional lymph nodes (lymph nodes along the lesser gastric curvature of post-operative day 7) of CD97/EGF1,2,5 kd group were demonstrated by immunohistochemistry, which indicated the potential involvement of CD97 in the preparation of (pre)metastatic niche (Figure 3D).

## Discussion

Previous studies demonstrated that CD97 isoforms play dual roles in gastric cancer cell migration and invasion, and only the CD97 small isoform is associated with increased invasive behavior of gastric cancer. To verify this theory, we generated stable knockdown clones of CD97 small isoform and established orthotopic gastric cancer mouse model with metastasis. We found in this study CD97 small isoform not only supported gastric cancer local growth, but also promoted metastatic spread in orthotopically implanted mouse model. Involvement of the CD97 small isoform in the preparation of (pre)metastatic niche could be one of the reasons.

It had been shown that CD97 protein was over-expressed in tumor tissues, especially in scattered tumor cells at the tumor invasion front, possessing much higher motile abilities than the tumor cells in the solid formation [20], [21]. Our previous studies revealed that strong co-expressions of CD97 and its ligand CD55 were exclusively localized at the tumor invasion front of gastric cancer, and additionaly correlated with its TNM status [14], [16]. These findings further support the participation of CD97 in gastric cancer cell migration and invasion. However, no relations with lymph node metastasis or lymphoid/blood vessel infiltration were found by clinicopathological investigations [15].

We demonstrated previously that over-expression of CD97/EGF1,2,5 or CD97/EGF1-5 in low CD97 cell line BGC-823 triggered the mechanisms resulting in quite different cell behaviours. The cells bearing CD97/EGF1,2,5 insert revealed increased invasive behavior, while the cells over-expressing CD97/EGF1-5 long isoform demonstrated tumor suppressive properties [19]. Other investigations concering CD97 variants revealed that over-expression of CD97/EGF1,2,5 insert propagated invasion of colon cancer cells, while C-terminal insertion reduced it. Besides, the motility of the cells remained almost unaffected when the full length isoform was over-expressed [17], what is with agreement to our findings. Thus, the CD97-induced augmentation of cancer invasiveness is closely related to its small isoform. Based on these observations and our previous findings, we focused further investigations on the specific role of CD97 small isoform in gastric carcinoma progression and metastasis.

SGC-7901 cells which expressed moderate levels of both CD97 small and big isoforms were selected as a suitable model to investigate the specific effects of CD97 small isoform. After knock-down of CD97/EGF1,2,5, the proliferative ability of CD97/EGF1,2,5 kd clones was significantly higher as compared to the wild-type or control groups, while the invasive ability was dramatically decreased. It conformed to our previous research that CD97 small isoform is associated with the invasive behavior, while the CD97 full length isoform is correlated with the proliferative property; and there existed an intrinsic balance between the two isoforms, when the expression of one isoform elevated, the other decreased. The elevation of the gelatinolytic abilities of CD97/EGF1,2,5 kd clones was considered due to the alternation of activity of MMPs. Galle J [17] reported HT1080 cells which over-expressing CD97 small isoform possessed the highest activity of matrix metalloproteinases (MMPs) MMP-2 and MMP-9, but HT1080 cells over-expressing CD97 full length isoform responded with almost unaltered or even decreased levels of MMPs as compared with wild-type or empty plasmid cells.

Although multivariate analysis reported no significant relations between CD97 expression and lymph node metastasis, results in this study showed noticeably retarded primary tumor growth as well as fewer metastatic tumor cells in regional lymph nodes in CD97/EGF1,2,5 kd group of the orthotopically transplanted metastatic mouse model of gastric carcinoma. This phenomenon conformed to the tumor invasive promoting role of CD97 small isoform. But how can CD97 small isoform facilitate lymph node metastasis?

In recent years, developed evidence based on Stephen Paget’s “seed and soil” hypothesis has emerged. Growth factors secreted by the primary tumor prime certain tissues for tumor cell engraftment [22]–[25]. In response to these soluble factors, tumor associated cells such as macrophages and haematopoietic progenitor cells cluster at some functional microenvironment, which is also known as “metastatic niches” that supports metastatic tumor cell maintenance and actively regulates cell proliferation and invasion [26]–[28]. This microenvironment is considered to comprise supportive stromal cells, soluble factors, vascular networks, nutrients and metabolic components, and the structural extracellular matrix (ECM) architecture [29]–[31].

VEGF, critical regulator of tumor angiogenesis, is thought to mobilize the bone marrow derived cells (BMDCs), which may subsequently be recruited and facilitate tumor growth and metastasis 888. It was reported that BMDCs express VEGFR localized to pre-metastatic sites before the arrival of tumor cells, and inhibition of VEGFR1 could prevent the BMDCs infiltration and “metastatic niche” formation in lungs [32], [33]. CD44 is also known to be essential for the homing and engraftment of the cancer stem cells [34]–[36]. It was reported after knocking down of CD44v6, it failed to assemble a soluble matrix in pre-metastatic organ, which allows a highly metastatic pancreatic cell line ASML embedding and growth. It strongly indicated both VEGFR and CD44 were involved in the preparation of (pre)metastatic niche [37].

In this study, VEGFR, CD44 along with platelet endothelial cell adhesion molecule (CD31) were together employed in an early lymph node metastatic model (post operative day 7) of gastric caner to investigate the possible mechanism of the metastasis-facilitating role of CD97 small isoform. In regional lymph nodes along the lesser gastric curvature prior to metastasis, the control group showed already existed, aggregated, high intensity of CD97 protein expression, as well as the elevated intensity of CD44, VEGF and CD31 expression; while the CD97/EGF1,2,5 kd clones showed comparatively scattered and down regulated transmembrane receptors protein expression, which indicated CD97 small isoform may also contribute to the metastasizing tumor cells settlement and involved in the preparation of the metastatic niche formation. However, information of how CD97 bearing tumor cells interacting with the metastatic organ surroundings and how the long-distance communication established is still lacking. Furthermore, it was reported the expression of CD97 was consistently suppressed in glioblastoma cell lines along with the silencing of WT1 [38], which suggested the possible upregulation of CD97 and its invasiveness promotion role were mediated by the regulation or expressional changes of other genes. But there was very little data describing the regulatory relationship of CD97 with other genes hitherto, which also merit further investigation.

In summary, taking advantage of the establishment of orthotopically transplanted metastatic mouse model of gastric carcinoma and the knocking down clones of CD97/EGF1,2,5 which showed poor metastatic ability, we demonstrate CD97 small isoform not only supports gastric cancer local growth, but also facilitates metastasis in a mouse model. Involvement in the preparation of the metastatic niche formation could be the reason for the contribution of CD97 small isoform to the metastasizing tumor cells settlement. Although exemplified in an animal model, the findings in this study are required to be controlled for their relevance in human cancer progression.

## Materials and Methods

### Cell Lines and Animals

All cell lines employed in this study were purchased from ATCC (www.atcc.org). Stomach adenocarcinoma cell line SGC-7901, MGC-801 and AGS cells were propagated in RPMI-1640 medium (Genom Biologic, Hangzhou); BGC-823 cells were propagated in Dulbecco’s minimal essential medium (DMEM)/Ham’s F12 (Genom Biologic, Hangzhou). All media were supplemented with 10% fetal bovine serum (FBS, Gibco) and 1% penicillin/streptomycin (Genom Biologic). The cells were grown in standard humidified incubator in 5% CO2 at 37°C and passaged every 4–7 days using trypsin-EDTA.

All the animal studies were carried out in strict accordance with the recommendations in the Guide for the Care and Use of Laboratory Animals of the National Institutes of Health and the protocol was approved by the Animal Research Committee of Zhejiang University, Hangzhou, China. All surgery was performed under sodium pentobarbital anesthesia, and all efforts were made to minimize suffering. Mouse protocols were conducted in accordance with stringent regulations laid out by Zhejiang University Laboratory Animal Center. Seventy-five 6–8-week old male BALB/c nu/nu mice weighing 18–22 g used for subcutaneous (15/75) or orthotopic tumor implantation (60/75) were randomly divided into control and CD97/EGF1,2,5 knockdown groups. Animals were housed in a sterile environment, cages and water were autoclaved, bedding and food was γ-ray-sterilized. All animals were maintained on daily 12-h light/12-h dark cycle, which was controlled by qualified staff in the Zhejiang University Laboratory Animal Center.

### Knockdown of CD97 Isoforms by RNA Interference (RNAi)

Four RNAi candidate target sequences of human CD97 isoform 3 (CD97/EGF1,2,5) were designed following the procedure of Dharmacon siDESIGN™ center (Table 1), and were cloned into pGCsilencer™ U6/Neo/GFP vector (Shanghai GeneChem) using Lipofectamine 2000 reagent according to manufacturer’s protocol (Life Technologies). Selection of the clones was initiated 2 days after transfection by employing 700 µg/ml of G418. Two weeks after transfection, positive clones were selected and maintained in fresh medium containing G418 at final concentration of 500 µg/ml. Medium was changed every 2–3 days and transfectants were passaged every 5–6 days. Expression of CD97 isoforms was verified by RT-PCR and Western blot analysis. Nonsilencing (NS)-siRNA was used as a control.

**Table 1:** RNAi candidate target sequences for CD97 isoform 3.

Sequence name	Sequence (5′-3′)
Nonsilencing-siRNA	TTCTCCGAACGTGTCACGT
CD97iso3-Si1	CCTGCATTCCAAGAAGCAA
CD97iso3-Si2	GCAGCTTTGCGATCCTTAT
CD97iso3-Si3	CATCCAGAATGTCATCAAA
CD97iso3-Si4	CTCAAACCTTGAAGATATC

### Orthotopic Transplantation of Human Gastric Cancer

Subconfluent SGC-NS (control) and SGC-CD97/EGF1,2,5 RNAi clone cells were harvested with trypsin-EDTA and resuspended to a final concentration of 1×10^8^/ml PBS. Hypodermic inoculation: 0.1 ml of cell suspension was subcutaneously injected into the right flank of respective mouse. Six-eight weeks later, when the size of tumor was around 1 cm^3^, tumor mass from each group was taken out and minced into pieces of approximately 1 mm^3^ for use in transplantation. Nude mice were anesthetized with pentobarbital sodium solution (Sinopharm Chemical Reagent, Beijing) via intraperitoneal injection (45 mg/kg). A left lateral laparotomy was performed. The stomach wall was carefully exposed, and a mechanical serosal injury was made in the middle of the greater curvature. One tumor piece was then fixed on the injured serosal surface with a 5–0 Dexon transmural suture.

### Evaluation of Growth and Metastasis of Orthotopically Implanted Tumors

To evaluate the growth and metastasis of orthotopically implanted tumors, mice were sacrificed according to the institutional guidelines on postoperative days 7, 14, 28 and 42 before they developed signs of distress. Autopsies were performed immediately, and primary tumors growing on the stomach wall were excised, weighed and examined histologically. The lungs, liver and enlarged regional or metastatic lymph nodes in peritoneal cavity were collected and processed for careful immunohistochemical examination.

### Total RNA Extraction and Reverse Transcriptase-polymerase Chain Reaction (RT-PCR)

Total RNA from SGC-7901 wild-type cells (SGCwt), stable SGC-NS and SGC-CD97/EGF1,2,5 RNAi clones was extracted using TRIzol reagent according to manufacturer’s instructions (Life Technologies). To exclude genomic amplification of prepro-CD97, specific intron-spanning oligonucleotide primers, which are suitable for amplification of all 3 CD97 isoforms (sense-actctgccgggagctgaaac; antisense-tggatggtgacctcggctga), were employed. RT-PCR reactions were performed in a 25-µl solution containing 4 µl of cDNA, 2.5 µl of 10× Advantage2 polymerase mix buffer, 10 nmol/l of dNTP, 20 pmol of the primer, and 2 U TaqDNA-polymerase (Life Technologies). PCR cycles consisted of an initial denaturation step for 5 min at 95°C, followed by 35 cycles of denaturation at 94°C for 30 sec, annealing for 45 sec at 61°C, elongation for 45 sec at 72°C, and a final extension cycle for 5 min at 72°C. PCR products were visualized on a 1% agarose gel containing 0.05% ethidium bromide. Semiquantitative RT-PCR analysis was performed with the Bio 1D software (LTF, Wasserburg, Germany) and β-actin served as normalizing marker.

### Cell Proliferation Assay (MTT Assay)

SGCwt, stable SGC-NS and SGC-CD97/EGF1,2,5 RNAi stable transfectants were plated in 200 µl of RPMI-1640 with 10% FBS at 2.5×10^4^cells/well in 96-well plates. After overnight incubation, the growth medium was replaced with serum-free medium. At each time-point (24, 48 and 72 h), 20 µl of 5 mg/ml MTT (Sigma) was added to each well and incubated at 37°C for 4 h to allow the reduction of MTT to formazan. Formazan crystals were dissolved in 100 µl DMSO and measured at 540 nm using ELISA reader (TECAN, Austria GmbH).

### Scratch-wound Assay

Cells were seeded at 2×10^5^ cells/well in 6-well plates and cultured as 70% confluent monolayers and deprived of serum for 24 h. A scratch was performed across the well with a standard 200 µl pipette tip and subsequently washed extensively with RPMI-1640 (supplemented with 1% FBS). The rate of scratch closure was determined by inverted phase contrast microscope (Leica, Germany) immediately and 24 h later. The migration rate was expressed as a percentage of the control (SGC-NS), and was calculated as the proportion of the mean distance between both borderlines caused by scratching to the distance that remained cell-free after re-growing. Two independent series of experiments were performed in quadruplicates.

### Soft Agar Colony Formation

Two-layered soft agar assays were performed in 6-well plates. The bottom layer of agar (1.5 ml per well) consisted of 2.5 ml of 3% agar (Roth) in sterile water, 1.5 ml of FBS, 150 µl of G418 (20 µg/ul), 150 µl of a 1∶1,000 dilution of amphotericin B (0.25 µg/ml; Sigma), and 450 µl of a 1∶1,000 dilution of gentamicin (10 µg/ml; Sigma) added to 15 ml with RPMI-1640 medium. Once solidified at room temperature for 10 min, 20,000 of SGCwt, SGC-NS and CD97/EGF1,2,5 RNAi clones were separately mixed into 1 ml of upper agar layer prepared from a stock consisting of 0.8 ml of 3% agar, 10% FBS, 75 µl of G418 (20 µg/ul), 75 µl (1∶1,000 dilution) of amphotericin B, 225 µl (1∶1,000 dilution) of gentamicin in 15 ml of culture medium. This cell suspension was carefully layered on the top of the bottom layer. Once the top agar layer had solidified, 1 ml of the culture medium was carefully added and changed once a week. After 4 weeks, cell colonies in the agar were stained overnight at 37°C in a 5% CO2 atmosphere with 200 µl of iodo-tetrazolium chloride (5 mg/ml; Sigma). Stained cell colonies were examined by light microscopy (Zeiss).

### Invasion and Migration Assays

The invasion assays were evaluated in 24-well Transwell™ chambers (Costar). The upper and lower culture compartments were separated by polycarbonate filters with 8 µm pore size. For invasion assay, the upper site of the filters (upper chamber) was coated with 1 mg/ml of gelatin (Sigma) before seeding the cells. SGCwt, SGC-NS and CD97/EGF1,2,5 RNAi clones were seeded at 2×10^4^cells/well in RPMI-1640 medium without FBS in the upper chamber and the lower chamber was filled with medium with 10% FBS. After 24-h incubation in a 5% CO2 atmosphere at 37°C, cells remaining on top of the filter were wiped off with cotton swabs and those transfectants that had traversed the membrane pores to the lower surface of the membrane were washed with chilled PBS, incubated for 5 min in 1∶1 PBS/methanol and 15 min in methanol before staining with 0.1% toluidine blue (Sigma) in 2.5% sodium carbonate. Migrated cells were counted by light microscopy (Zeiss) in four separate high-power fields per filter. For the migration assay, subconfluent monolayer was scratched with a pipette tip. Wound healing was evaluated after 24–72 h by light microscopy. All experiments were performed at least in triplicates and were expressed as mean±SEM.

### Western Blot Analysis

CD97 protein expression was analyzed by western blot analysis. SGCwt, SGC-NS and CD97/EGF1,2,5 RNAi clones were seeded at 1.5×10^5^/well in 25 cm^2^ flasks and cultured in medium with 10% FBS until 70% of confluency. Total proteins were isolated with 2× extraction buffer (125 mM Tris-HCl pH 6.8; 4% sodium dodecylsulfate (SDS); 20% glycerol; 10% mercaptoethanol (ME); 2% bromophenol blue) plus protease inhibitors cocktail (all reagents from Sigma). Proteins were resolved on a 12% SDS gel, blotted at 1 mA/gel cm2 for 2 h onto Hybond-ECL nitrocellulose membranes (Amersham). Membranes with blotted proteins were blocked for 2.5 h with 5% milk in PBS plus 0.02% Tween-20 (PBST; Sigma) and incubated overnight at 4°C with the mouse polyclonal CD97 antibody (1∶5,000; Abnova). Secondary HRP-conjugated rabbit anti-mouse IgGs was used at 1∶20,000 for 1 h at RT. Immunoreactive protein bands were visualized with the ECL kit (Amersham). The same membranes were reprobed with mAbs specific to human β-actin prior to incubation in stripping solution (2% SDS; 125 mM Tris-HCl, pH 8.0; 0.7% ME) and the block buffer (5% milk in PBS plus 0.02% Tween-20). β-actin was visualized with secondary goat anti-mouse antibodies (Sigma, 1∶20,000 in blocking buffer for 1 h) and ECL-kit.

### Flow Cytometry

Lymph nodes (LN) aseptically removed were cut into small pieces and meshed through fine gauze. Suspended cells (2–5×10^5^) were stained with fluorochromeconjugated mAbs against C4.4A (Santa Cruz Biotechnology) according to routine procedures. For intracellular staining, cells were fixed and permeabilized using Cytofix/Cytoperm (BD PharMingen) according to the manufacturer’s instructions. The number of tumor cells (C4.4A +) in LN was evaluated by LSR II flow cytometer (Becton Dickinson, San Jose, CA), and data were analyzed using Flowjo software (Tree Star, Inc., Ashland, OR).

### Immunohistochemistry

Immunohistochemistry was performed on frozen sections using the AEC Chromogen Kit (Sigma) according to manufacturer’s protocol. Frozen tissues were sectioned (5–7 µm thickness), mounted and air-dried. After having been fixed in cold acetone and washed with PBS, the sections were incubated in 0.03% hydrogen peroxide for 15 min to inactivate endogenous peroxidase. Slides were then incubated with antibodies against mouse CD97 (1∶200; Abnova), CD44 (1∶200, Abcam), CD31(1∶200, Abcam) and VEGF(1∶400, Abcam). Positive reactions (rose-red insoluble precipitates) were developed by incubating the slides with AEC substrate reagent after treatment with the corresponding peroxidase-conjugated secondary antibodies. The sections were then counter-stained with Mayer’s hematoxylin (invitrogen).

### Statistical Analysis

Statistical analysis was performed with the SPSS 10.0 software. Student’s t-test and one-way analysis of variance were used. All experiments were performed at least in triplicates and were expressed as mean±SEM with P-values of <0.05 considered as statistically significant.

## References

[1] van Lier RA, Eichler W, Hamann J (1996). Sevenspan transmembrane molecules: novel receptors involved in leukocyte adhesion.. Immunol Lett.

[2] Hamann J, Hartmann E, van Lier RA (1996). Structure of the human CD97 gene: exon shuffling has generated a new type of seven-span transmembrane molecule related to the secretin receptor superfamily.. Genomics.

[3] McKnight AJ, Gordon S (1996). EGF-TM7: a novel subfamily of seven-transmembrane-region leukocyte cell-surface molecules.. Immunol Today.

[4] Gray JX, Haino M, Roth MJ, Maguire JE, Jensen PN (1996). CD97 is a processed, seven-transmembrane, heterodimeric receptor associated with inflammation.. J Immunol.

[5] Spendlove I, Ramage JM, Bradley R, Harris C, Durrant LG (2006). Complement decay accelerating factor (DAF)/CD55 in cancer.. Cancer Immunol Immunother.

[6] Mikesch JH, Schier K, Roetger A, Simon R, Buerger H (2006). The expression and action of decay-accelerating factor (CD55) in human malignancies and cancer therapy.. Cell Oncol.

[7] Martens GJ (1992). Molecular biology of G-protein-coupled receptors.. Prog Brain Res.

[8] Eichler W, Aust G, Hamann D (1994). Characterization of an early activation-dependent antigen on lymphocytes defined by the monoclonal antibody BL-Ac(F2).. Scand J Immunol.

[9] Steinert M, Wobus M, Boltze C, Schütz A, Wahlbuhl M (2002). Expression and regulation of CD97 in colorectal carcinoma cell lines and tumor tissues.. Am J Pathol.

[10] Hoang-Vu C, Bull K, Schwarz I, Krause G, Schmutzler C (1999). Regulation of CD97 protein in thyroid carcinoma.. J Clin Endocrinol Metab.

[11] Jaspars LH, Vos W, Aust G, Van Lier RA, Hamann J (2001). Tissue distribution of the human CD97 EGF-TM7 receptor.. Tissue Antigens.

[12] Aust G, Eichler W, Laue S, Lehmann I, Heldin NE (1997). CD97: a dedifferentiation marker in human thyroid carcinomas.. Cancer Res.

[13] Inoue T, Yamakawa M, Takahashi T (2002). Expression of complement regulating factors in gastric cancer cells.. Mol Pathol.

[14] Liu Y, Chen L, Peng SY, Chen ZX, Hoang-Vu C (2005). Role of CD97(stalk) and CD55 as molecular markers for prognosis and therapy of gastric carcinoma patients.. J Zhejiang Univ Sci B.

[15] Aust G, Steinert M, Schütz A, Boltze C, Wahlbuhl M (2002). CD97, but not its closely related EGF-TM7 family member EMR2, is expressed on gastric, pancreatic, and esophageal carcinomas.. Am J Clin Pathol.

[16] Liu Y, Chen L, Peng S, Chen Z, Gimm O (2005). The expression of CD97EGF and its ligand CD55 on marginal epithelium is related to higher stage and depth of tumor invasion of gastric carcinomas.. Oncol Rep.

[17] Galle J, Sittig D, Hanisch I, Wobus M, Wandel E (2006). Individual cell-based models of tumor-environment interactions: Multiple effects of CD97 on tumor invasion.. Am J Pathol.

[18] Hensel F, Hermann R, Brändlein S, Krenn V, Schmausser B (2001). Regulation of the new coexpressed CD55 (decay-accelerating factor) receptor on stomach carcinoma cells involved in antibody SC-1-induced apoptosis.. Lab Invest.

[19] Liu D, Trojanowicz B, Radestock Y, Fu T, Hammje K (2010). Role of CD97 isoforms in gastric carcinoma.. Int J Oncol.

[20] Park KJ, Choi HJ, Roh MS, Kwon HC, Kim C (2005). Intensity of tumor budding and its prognostic implications in invasive colon carcinoma.. Dis Colon Rectum.

[21] Nakanishi Y, Ochiai A, Kato H, Tachimori Y, Igaki H (2001). Clinicopathological significance of tumor nest configuration in patients with esophageal squamous cell carcinoma.. Cancer.

[22] Weiss L, Mayhew E, Rapp DG, Holmes JC (1982). Metastatic inefficiency in mice bearing B16 melanomas.. Br J Cancer.

[23] Hiratsuka S, Watanabe A, Aburatani H, Maru Y (2006). Tumour-mediated upregulation of chemoattractants and recruitment of myeloid cells predetermines lung metastasis.. Nat Cell Biol.

[24] Zöller M (2009). Tetraspanins: push and pull in suppressing and promoting metastasis.. Nat Rev Cancer.

[25] Kaplan RN, Rafii S, Lyden D (2006). Preparing the ‘soil’: the premetastatic niche.. Cancer Res.

[26] Li L, Neaves WB (2006). Normal stem cells and cancer stem cells: the niche matters.. Cancer Res.

[27] Yin T, Li L (2006). The stem cell niches in bone.. J Clin Invest.

[28] Zhang J, Li L (2008). Stem cell niche: microenvironment and beyond.. J Biol Chem.

[29] Folkman J (2002). Role of angiogenesis in tumor growth and metastasis.. Semin Oncol.

[30] Weigelt B, Bissell MJ (2008). Unraveling the microenvironmental influences on the normal mammary gland and breast cancer.. Semin Cancer Biol.

[31] Joyce JA, Hanahan D (2004). Multiple roles for cysteine cathepsins in cancer.. Cell Cycle.

[32] Psaila B, Lyden D (2009). The metastatic niche: adapting the foreign soil.. Nat Rev Cancer.

[33] Kaplan RN, Riba RD, Zacharoulis S, Bramley AH, Vincent L (2005). VEGFR1-positive haematopoietic bone marrow progenitors initiate the pre-metastatic niche.. Nature.

[34] Zöller M (2011). CD44: can a cancer-initiating cell profit from an abundantly expressed molecule?. Nat Rev Cancer.

[35] Nie D (2011). Cancer stem cell and niche.. Front Biosci (Schol Ed).

[36] Iwasaki H, Suda T (2009). Cancer stem cells and their niche.. Cancer Sci.

[37] Jung T, Castellana D, Klingbeil P, Cuesta Hernández I, Vitacolonna M (2009). CD44v6 dependence of premetastatic niche preparation by exosomes.. Neoplasia.

[38] Chidambaram A, Fillmore HL, Van Meter TE, Dumur CI, Broaddus WC (2012). Novel report of expression and function of CD97 in malignant gliomas: correlation with Wilms tumor 1 expression and glioma cell invasiveness.. J Neurosurg.

